# Diffusion Tensor Tractography Studies on Recovery Mechanisms of Aphasia in Stroke Patients: A Narrative Mini-Review

**DOI:** 10.3390/healthcare10101927

**Published:** 2022-09-30

**Authors:** Sung Ho Jang, Sang Seok Yeo, Eun Bi Choi

**Affiliations:** 1 Department of Physical Medicine and Rehabilitation, College of Medicine, Yeungnam University, 317-1, Daemyungdong, Namku, Daegu 705-717, Korea; 2 Department of Physical Therapy, College of Health Sciences, Dankook University, 119, Dandae-ro, Dongnam-gu, Cheonan-si 330-714, Korea

**Keywords:** diffusion tensor imaging, diffusion tensor tractography, stroke, aphasia, recovery

## Abstract

Aphasia is a common and serious clinical feature of stroke. Various neural tracts are known to be involved in language processing. Diffusion tensor tractography (DTT) appears to be an appropriate imaging technique for the elucidation of the recovery mechanisms of aphasia in the language-related neural tracts in stroke patients. In this article, twelve previous DTT-based studies on the recovery mechanisms of aphasia in stroke were reviewed. We classified the twelve studies into the following three categories according to the recovery mechanisms: recovery via the neural tracts in the dominant hemisphere (eight studies), via transcallosal fibers (two studies), and via the neural tracts in the non-dominant hemisphere (two studies). Although there are various neural tracts for language processing, eight of the ten studies focused only on the role of the arcuate fasciculus (AF) in the recovery process. Consequently, it appears from the studies that only one recovery mechanism of aphasia via the restoration of the integrity of the injured AF in the dominant hemisphere was clearly demonstrated. However, because various neural tracts are involved in language processing, there could be other mechanisms that have not yet been elucidated. Therefore, further original studies involving a larger number of patients with aphasia in stroke should be encouraged forthwith. Further studies involving various lesion locations and severity levels of injuries to the language-related neural tracts are also necessary because the recovery mechanisms of aphasia in stroke could be dependent on these factors.

## 1. Introduction

Aphasia is the inability to comprehend or formulate language because of damage to specific regions of the brain which are related to language function [1]. Aphasia is a common and serious clinical feature of stroke: approximately 30% of stroke patients have been reported to present with aphasia at the acute stage, whereas 10~18% of stroke patients are reported to have aphasia as sequelae at the chronic stage [2,3,4,5,6,7]. As a result, aphasia in stroke can pose a significant burden for patients and caregivers. The elucidation of the mechanisms of aphasia recovery in stroke is clinically important and essential for establishing effective neuro-rehabilitation strategies. More specifically, novel non-invasive brain stimulation therapies such as repetitive transcranial magnetic stimulation (rTMS) or transcranial direct current stimulation (tDCS) can be applied to specific neural structures which contribute to aphasia recovery [7,8,9,10]. 

Functional neuroimaging studies, including functional magnetic resonance imaging (fMRI) and nuclear medicine imaging, have revealed a few recovery mechanisms. These include the activation of the peri-stroke area, restoration of the language-related network in the dominant hemisphere, and activation of the homologous language areas in the nondominant hemisphere [8,11,12,13,14,15,16,17]. Functional neuroimaging techniques have the advantage of detecting the changes in the specific regions of the brain, especially the cerebral cortex, which are involved in language processing during the recovery of aphasia. However, these have a limitation in identifying the changes in the language-related neural tracts at the subcortical level during this phase. 

By contrast, the recently developed diffusion tensor tractography (DTT), derived from diffusion tensor imaging (DTI), has a unique advantage due to its ability to visualize and estimate the language-related neural tracts at the subcortical level [18,19,20,21,22,23,24,25,26,27,28,29,30]. The main advantage of DTT is that the entire neural tract can be evaluated using the DTT parameters (fractional anisotropy [FA], mean diffusivity, and fiber number) and configurational analysis [31,32,33]. As a result, serial DTTs for the language-related neural tracts during the improvement phase appear to be appropriate for the elucidation of the mechanisms of aphasia recovery in them [8,18,19,21,22,23,24,25,26,27,28,29,30,34,35,36]. A significant number of DTT-based studies have reported on the recovery mechanisms of aphasia in various brain pathologies including stroke, traumatic brain injury, and brain tumor [8,18,19,21,22,23,24,25,26,27,28,29,30,34,35,36]. The majority of the above studies have reported on stroke patients with aphasia [18,19,21,22,23,24,25,26,27,28,29,30]. However, there has been no review article on this topic in stroke.

This review aims to present an in-depth analysis of the DTT-based studies that have reported the mechanisms of aphasia recovery in stroke patients by demonstrating the changes in the language-related neural tracts. 

## 2. Neural Tracts Which Are Involved in Language Processing

**Figure 1:**
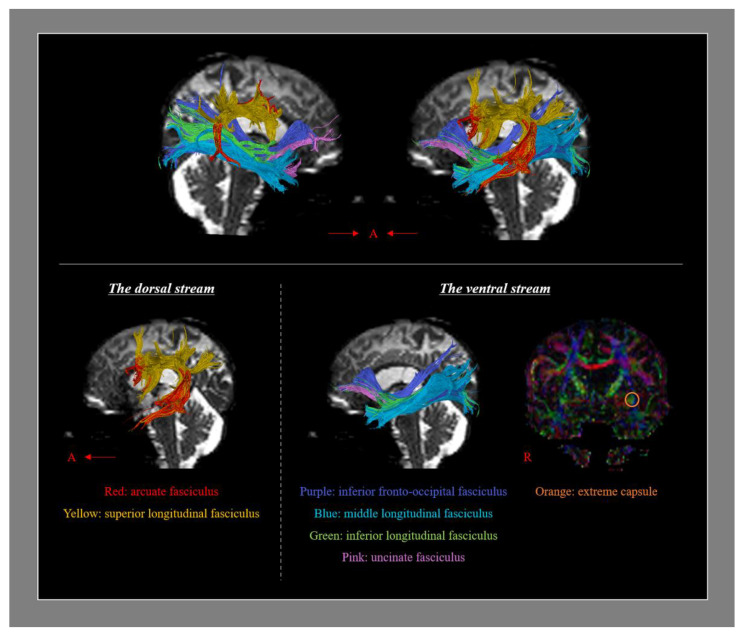
Diffusion tensor tractography for the neural tracts which are involved in language processing (A: anterior, R: right). The dorsal stream contains the arcuate fasciculus and the superior longitudinal fasciculs, and the ventral stream contains the inferior fronto-occipital fasciculus, the middle longitudinal fasciculus, the inferior longitudinal fasciculus, and the uncinate fasciculus.

Various neural tracts are known to be involved in language processing [37,38,39,40,41,42,43,44,45]. The dual stream model suggests that the neural tracts can be classified into two categories: the dorsal and the ventral stream, wherein the dorsal stream integrates sensorimotor processing for phonation and the ventral stream mediates comprehension. The dorsal stream connects the temporoparietal and frontal premotor regions via the arcuate fasciculus (AF) and superior longitudinal fasciculus (SLF, especially SLF III), and the ventral stream connects the temporal and prefrontal regions via the inferior fronto-occipital fasciculus (IFOF), middle longitudinal fasciculus (MLF), inferior longitudinal fasciculus (ILF), uncinate fasciculus (UF), and extreme capsule (Figure 1) [37,38,39,40,41,42,44,45]. The AF connecting the Broca’s and Wernicke’s areas is the major fiber tract of the dorsal stream, while the IFOF connecting the frontal lobe and the temporobasal areas, superior parietal lobe, and occipital cortex is the major tract of the ventral stream [41]. It is essential to know the precise role of each neural tract for language processing in the mechanisms of aphasia recovery, yet the roles have not been clearly elucidated in research thus far [41,45]. A recent systemic review has elaborated on the functions of each of the language-related neural tracts: the left IFOF predominated across most linguistic aspects in terms of global severity, comprehension, naming, and reading ability. The left UF and ILF had a significant correlation with comprehension and the left AF and SLF III were significantly correlated with syntactic processing [45]. The correlations with the language subdomains were as follows; naming (the left IFOF: *r* = 0.56; the left UF: *r* = 0.49; and the left ILF: *r* = 0.35), repetition (the left AF: *r* = 0.48), syntactic processing (the left AF: *r* = 0.61; and the left SLF: *r* = 0.55); reading (the left IFOF: *r* = 0.34; and the left UF: *r* = 0.28); and comprehension (the left IFOF: *r* = 0.53; the left UF: *r* = 0.38; and the left ILF: *r* = 0.48) (Table 1) [45]. On the other hand, the left frontal aslant tract which connects the left lateral inferior frontal gyrus with the pre-supplementary and supplementary motor areas is known to be involved in speech initiation and verbal fluency [44].

## 3. The Useful Characteristics of DTT for Research on the Mechanisms of Aphasia Recovery in Stroke Patients

DTT has been used for the determination of the state of a neural tract using the DTT parameters, namely FA, mean diffusivity, fiber number (voxel number and tract volume), and configurational analysis (partial tearing, narrowing, or discontinuation) [31,32,33,46,47,48]. The FA value which is the most widely used parameter indicates the state of white matter organization by reflecting the degree of directionality and integrity of the subcortical white matter microstructures [30,31,32,33,46,47,48]. The FA value can increase with the increased organization of a neural tract (for example, recovery of an injured neural tract), whereas it can decrease with the disintegration of a neural tract (for example, injury of a neural tract). The mean diffusivity value suggests the magnitude of water diffusion, which can increase with some pathological conditions such as vasogenic edema or axonal injury [30,31,32,33,46,47,48]. By contrast, the fiber number of a neural tract, which is a measure of the number of voxels contained within a neural tract, indicates the number of neural fibers within it. In addition, the configurational analysis provides information on the integrity and configuration of the neural tract and has been used as a biomarker to indicate the state of the neural tract [18,33,49]. Therefore, the recovery mechanisms of aphasia can be determined based on the changes in the DTT parameters and configuration of the language-related neural tracts on serial DTTs and can be matched concurrently with the improvement in aphasia. Even though the DTT reconstruction method has been reported to be highly reliable [50], some limitations of the procedure should be considered. DTT may misestimate the neural tracts due to regions of fiber complexity and crossing fibers that can inhibit the complete reconstruction of the real fiber architecture [51,52,53]. Therefore, the precise analysis of the neural tracts on DTT requires the availability of experienced researchers.

## 4. Review of DTT Studies on the Mechanisms of Aphasia Recovery in Stroke Patients

**Figure 2:**
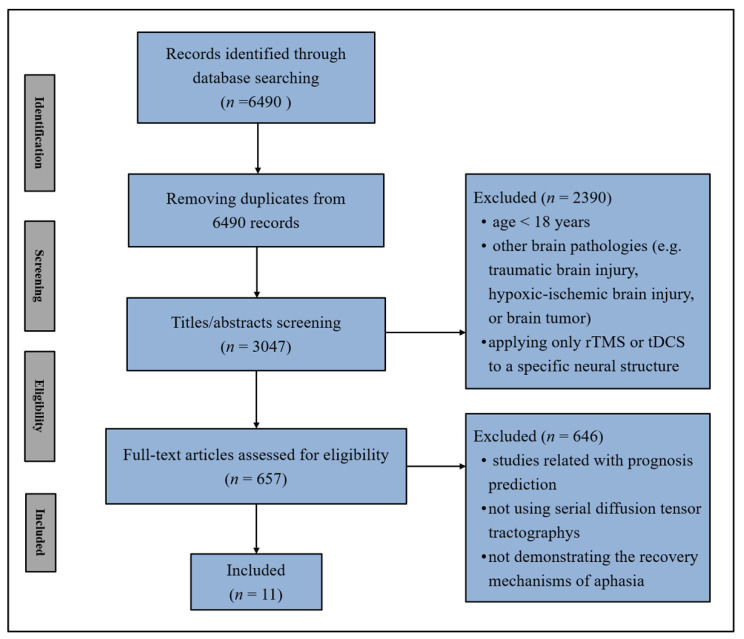
Flow diagram of the recovery mechanisms of aphasia study selection (rTMS: repetitive transcranial magnetic stimulation, tDCS: transcranial direct current stimulation).

A literature search for DTT-based studies elucidating the mechanisms of recovery from aphasia in stroke patients was conducted for the period January 1992 to July 2022, using electronic databases (MEDLINE database [PubMed], Web of Science, Google Scholar, and ScienceDirect). The search strategy for identifying potentially relevant articles was based on the subject heading and keywords/abbreviations with synonyms as follows: (“diffusion tensor imaging” OR “diffusion tensor tractography” OR DTI OR DTT) AND (“stroke” OR “cerebral infarction” OR “intracerebral hemorrhage”) AND (“aphasia”) AND (“recovery” OR “plasticity”). We confined ourselves to the studies in which the recovery mechanisms of aphasia in the language-related neural tracts were demonstrated through serial DTTs in adult stroke patients and excluded the studies which applied only rTMS or tDCS to a specific neural structure. Overall, twelve studies were selected for review (Figure 2, Table 2) [18,19,21,22,23,24,25,26,27,28,29,30]. According to the recovery mechanisms of aphasia, we classified the twelve studies into the following three categories: recovery via the neural tracts in the dominant hemisphere (eight studies), via transcallosal fibers (two studies), and via the neural tracts in the non-dominant hemisphere (two studies) (Table 2).

### 4.1. Recovery via the Neural Tracts in the Dominant Hemisphere

In 2011, Breier et al. reported the case of a chronic stroke patient who presented with a change of the left AF concurrent with an improvement of aphasia [19]. The 62-year-old male patient had suffered aphasia due to an infarction in the left middle cerebral artery territory (all of the left frontal lobe, insula, and subcortical areas including the internal capsule) five years previously. The patient underwent constraint-induced language therapy (CILT), which is speech therapy for aphasia based on the principles of use-dependent learning, for three weeks (three hours/day and four days/week) [54]. Aphasia evaluation, DTI, and magneto-encephalography (MEG) were performed four times: three weeks prior to, immediately prior to, immediately after, and three months after CILT. On evaluation, the aphasia quotient (AQ) of the Western Aphasia Battery (WAB) improved as follows with CILT: three weeks pre-CILT (59.2 percentile), immediately pre-CILT (56.2 percentile), immediately post-CILT (66.8 percentile), and 3 months post-CILT (68.2 percentile) [19]. Prior to the CILT program, high activations were observed in the left posterior superior temporal gyrus on both pre-CILT MEGs. After the CILT program, activation was detected in the left temporal lobe on both post-CILT MEGs. On DTT, the FA value of the left AF increased immediately after the CILT program and increased further three months after the CILT program ([the approximate FA value] pre-CILT: 0.36 → immediately post-CILT: 0.37 → 3 months post-CILT: 0.39; the precise FA values were not described), whereas the FA values of the right AF remained stable irrespective of the CILT program (approximately 0.4). In addition, the presence of transcallosal fibers between both AFs through the splenium of the corpus callosum was observed immediately after the CILT program; however, these disappeared three months after the CILT program. The authors concluded that these results provide evidence of an association between the improvement of aphasia and the increased integrity of the left AF by speech therapy in this patient. This was the first study to demonstrate that the increased integrity of the left AF is related to the improvement of aphasia in stroke patients. Furthermore, the authors tried to demonstrate the immediate and long-term effect of speech therapy by administering DTT four times in a chronic stroke patient with aphasia. However, this data had the limitation of being a case study that was performed without control subjects [19].

In 2014, Hee et al. investigated the changes in the left AF following speech therapy in chronic stroke patients with aphasia [22]. Eight right-handed patients who had suffered aphasia due to a stroke in the left hemisphere, 17 to 170 months after onset (detailed stroke pathologies and lesion locations were not described) underwent four weeks of speech therapy (three sessions/week). Semantic or phonological therapy was used alternately in each session, and the order was counterbalanced among the patients [55,56]. After the speech therapy program, six of the eight patients showed an improvement in aphasia on the Boston Naming Test (BNT) [57]. DTI scanning was done twice (prior to and post-speech therapy programs). The FA value of the left AF was observed to be significantly increased on the post-therapy DTT compared to the pre-therapy DTT without a significant change in the fiber number. In contrast, no significant changes in the FA value and fiber number were detected in the right AF and both UFs. The authors concluded that speech therapy appeared to improve the integrity of the left AF in these chronic stroke patients with aphasia. The advantage of this study was that the authors analyzed the UF which is related to the semantic process as well as the AF [22]. However, the number of subjects was relatively small and there was no control group. In addition, the patients were heterogeneous in terms of their aphasia types (anomic: six patients and conduction: two patients) and severity (mild: five patients, mild to moderate: one patient, and moderate: two patients).

**Figure 3:**
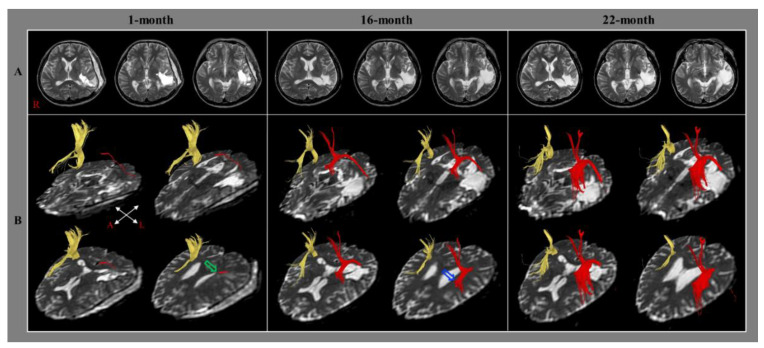
(**A**) Brain MR images at 1, 16, and 22 months after onset show leukomalatic lesions in the left parieto-temporal areas (R: right). (**B**) DTT for the arcuate fasciculus (right AF, yellow; left AF, red). The right AF passes through the known pathway of the AF. On 1-month DTT, the left AF showed discontinuation (green arrow) after originating from the Wernicke area. In contrast, on 16-month DTT, the injured left AF was thickened and elongated to around the left Broca area; however, discontinuation (blue arrow) of the left AF was observed around the left Broca area without termination at the left frontal area. On 22-month DTT, the discontinuation of the left AF was elongated to the left Broca area and the left AF was thickened (reprinted with permission from Ref. [21]).

In 2014, Jang and Lee reported the case of a stroke patient who presented with a change of injured left AF concurrent with an improvement of aphasia [21]. The 43-year-old right-handed male patient was diagnosed with a spontaneous intracerebral hemorrhage and received treatment involving the removal of hematoma after decompressive craniectomy. Brain MRIs taken at one month after onset revealed leukomalactic lesions in the left parieto-temporal areas (Figure 3A). The patient showed severe aphasia on the AQ of the WAB (5 percentile) one month after onset [58]. He underwent comprehensive rehabilitative therapy for one month, including speech therapy with neurotropic drugs (methylphenidate, pramipexole, and amantadine) at a university hospital, and his aphasia mildly improved to 19 percentiles on the AQ of the WAB. Subsequently, he went through a similar rehabilitation program including speech therapy at a local rehabilitation hospital for nine months after onset. He underwent speech therapy (once a week) with similar medications at the outpatient clinic of the university hospital until 22 months after onset. His aphasia was improved to 42 and 58 percentiles on the AQ of WAB at 16 months and 22 months after onset, respectively. On the 1-month DTT, only the thin ascending part of the left AF from the Wernicke’s area was observed. In contrast, on the 16-month DTT, the injured left AF was thickened and elongated to around the left Broca’s area without termination in the left frontal area. However, the left AF was elongated to the left Broca’s area and was thickened on the 22-month DTT also (Figure 3B). Thus, using serial language tests and DTTs, the authors demonstrated that the improvement of aphasia was related to the recovery of the injured left AF for approximately two years from the early to the chronic stage of stroke. However, the authors provided only configurational data of the left AF without DTT parameters. In addition, this study has the limitation of being a case report and there is a lack of data on other language-related neural tracts [21].

During the same year, Nunnari et al., 2014 reported the case of a stroke patient who showed an increase in integrity of the fibers of the left AF along with an improvement in language function after rehabilitation [23]. The 49-year-old right-handed female patient was diagnosed with motor aphasia due to ischemic stroke in the left temporoparietal area and underwent rehabilitation including speech therapy for one month. The Neuropsychological Examination of Aphasia Battery presented the presence of deficits in different subtests before rehabilitation. However, a global cognitive recovery with improvements of the verbal and written communications were observed after rehabilitation. On the second DTT, the FA value and fiber number of the left AF were increased without change in the right AF after rehabilitation. As a result, the authors concluded that the increased integrity of the left AF after rehabilitation appeared to be related to language recovery in this patient [23]. However, this study is limited as a case report and the authors did not precisely describe when rehabilitation was performed after stroke onset.

Subsequently, Jang et al., 2017 investigated the relationship between the improvement of aphasia and the alterations in the injured left AF in stroke patients. Sixteen right-handed stroke patients with aphasia and left AF injury were recruited (cerebral infarction: 11 patients and intracerebral hemorrhage: five patients) [24]. The WAB and DTTs for the AF were performed twice, within 30 days and three months after onset [58]. A positive correlation was observed between the improvement of the AQ of the WAB and the increased voxel number of the left AF (*r =* 0.626). However, no correlation was detected between the improvement of the AQ and changes in the FA value of the left AF, and the voxel number and FA value of the right AF. These results indicated that the improvement of aphasia in these patients was associated with the recovery of the injured left AF, irrespective of the directionality of the left AF from the early to chronic stages of stroke. Consequently, the authors suggested that the facilitation of the recovery of the injured left AF could be an important strategy in the neurorehabilitation of stroke patients with aphasia. For example, non-invasive neurostimulation such as rTMS can be applied to facilitate recovery of the injured left AF irrespective of the effects of the inhibition of the right AF [24]. However, this study is limited due to the small number of subjects. Furthermore, this study did not consider the state of the left AF. It is probable that the recovery mechanism of aphasia could be different depending on the state of the left AF. Also, other than AF, no other language-related neural tracts were analyzed in the study [24].

In 2021, Kierońska et al. reported a stroke patient who presented with changes in the AF and UF concurrent with an improvement of aphasia [29]. The 41-year-old, right-handed male patient was diagnosed with cerebral infarction in the left temporal lobe. The patient presented with total sensory and partial motor aphasia scoring 0/30 points on the Frenchay Aphasia Screening Test (FAST) which estimates comprehension, expression, reading, and writing, and 2/60 points on the BNT which measures naming skills [57,59]. The patient underwent conventional rehabilitation (exercises with a physiotherapist) and received transcranial direct current stimulation (tDCS) during the first ten days after onset. The tDCS was administered for 30 min daily with 2 mA amplitude (anodal tDCS: the left hemisphere language areas to increase cortical excitability and cathodal tDCS: the right hemisphere homotopic areas to inhibit overactivation in the contralesional right homologs of the language areas). The patient showed progressive improvements of aphasia, scoring 20/30 points in the FAST, 45/60 points in the BNT at ten days after onset, and 29/30 points in the FAST and 60/60 points in the BNT at three months after onset. Increments in the fiber number and tract volume were observed in both the AF and the left UF on the 3-month DTT compared to the admission-day DTT. The authors concluded that the improvement of aphasia was associated with changes in the fiber number and tract volume of the AF and UF. The authors attempted to investigate the changes in the AF and UF using DTT during the improvement of aphasia. However, DTI data at the acute stage of cerebral infarction can be unstable and could be affected by local factors such as peri-infarct edema [60,61]. In addition, the patient underwent tDCS for the first ten days. However, there were no control patients with similar lesions.

During the same year, Choi et al., 2021 reported a stroke patient whose aphasia improved along with the restoration of the injured left AF following cranioplasty [27]. The 57-year-old, right-handed male patient received decompressive craniectomy in the left fronto-parieto-temporal areas along with hematoma removal for intracerebral hemorrhage and subarachnoid hemorrhage. He exhibited severe aphasia at 20.7 percentile on the AQ of the WAB at eight weeks after onset (one week before cranioplasty) and received cranioplasty at nine weeks after onset. At two weeks after cranioplasty, he presented with a significant improvement of aphasia at 44.7 percentile on the AQ of WAB [58]. The left AF that originated from the Wernicke’s area was disrupted around the Broca’s area on the pre-cranioplasty DTT. In contrast, the left AF was elongated and connected to the left Broca’s area on the post-cranioplasty DTT. The FA value of the left AF decreased from 0.430 to 0.392 after cranioplasty, while the fiber number increased from 760 to 960. Consequently, the authors concluded that the restoration of the curved cerebral cortex following cranioplasty contributed to the restoration of the injured left AF in this patient. The advantage of this study was that it provided the changes of the DTT parameters with the configurational change of the left AF. However, a limitation of this study was that there was no reconstruction of other language-related neural tracts.

Recently, Bae et al., 2022 investigated the relationship between the change of aphasia and the changes of both AFs in stroke patients [30]. Thirty-five right-handed stroke patients with aphasia were recruited (cerebral infarction: 19 patients and intracerebral hemorrhage: 16 patients). The WAB and DTTs for the AF were performed twice (the WAB; first; average 29.40 days after onset and second; average 181.77 days after onset). They found that the FA values of both AFs decreased and the diffusivity increased at the chronic stage than those at the subacute stage of stroke. However, a positive correlation was observed between the change of the AQ of the WAB and the change of the FA value of the left AF (*r =* 0.365), whereas no correlation was observed in the right AF. Furthermore, the patient group with increased FA value of the left AF (12 patients) showed more significant increment of the AQ than the patient group with decreased FA value (23 patients). As a result, the authors concluded that the integrity of AF decreased in both hemispheres in patients with aphasia from the subacute to chronic stage of stroke, and the change in structural connectivity of the left AF (increased FA value) was associated with aphasia improvement. Their findings were helpful to understand the natural course of structural connectivity changes in the language network in aphasia patients in the subacute phase after stroke and the underlying mechanisms of language recovery [30].

### 4.2. Recovery via Transcallosal Fibers

In 2018, Yu et al. reported the case of a chronic stroke patient who showed changes in the transcallosal fibers to the cortical language areas concurrent with an improvement of aphasia by speech therapy [25]. The 33-year-old right-handed male patient presented with aphasia due to an infarction in the left frontal lobe, insula, and basal ganglia which had occurred 14 months previously. During the subsequent five months, the patient underwent speech therapy which consisted of spontaneous speech, auditory comprehension, repetition, naming, writing, reading training, and calculation (1 h/session, twice/day, and five days/week). His aphasia revealed certain improvements on a modified Chinese WAB after a speech therapy program compared to his score prior to the speech therapy program (spontaneous speech: 69.8 percentile to 81.1 percentile, auditory comprehension: 66.4 percentile to 69.4 percentile, repetition: 64.0 percentile to 72.0 percentile, and naming: 84.8 percentile to 84.8 percentile) [62]. On the post-speech therapy DTT for the corpus callosum, the fiber pathway between the splenium of the corpus callosum and the left superior temporal gyrus (Wernicke’s area) had been newly reconstructed and the fiber connections between the genu of the corpus callosum and the right inferior frontal gyrus (the mirror region of Broca’s area) were increased compared to the pre-speech therapy DTT. Consequently, the authors inferred that the changes between the corpus callosum and cortical language areas after stroke could contribute to the improvement of aphasia in stroke patients. To the best of our knowledge, this was the first study to suggest that the transcallosal fibers connected to the cortical language areas can contribute to aphasia recovery except for naming. However, as with previous studies, in this report too, no analysis was carried out on the other language-related neural tracts.

**Figure 4:**
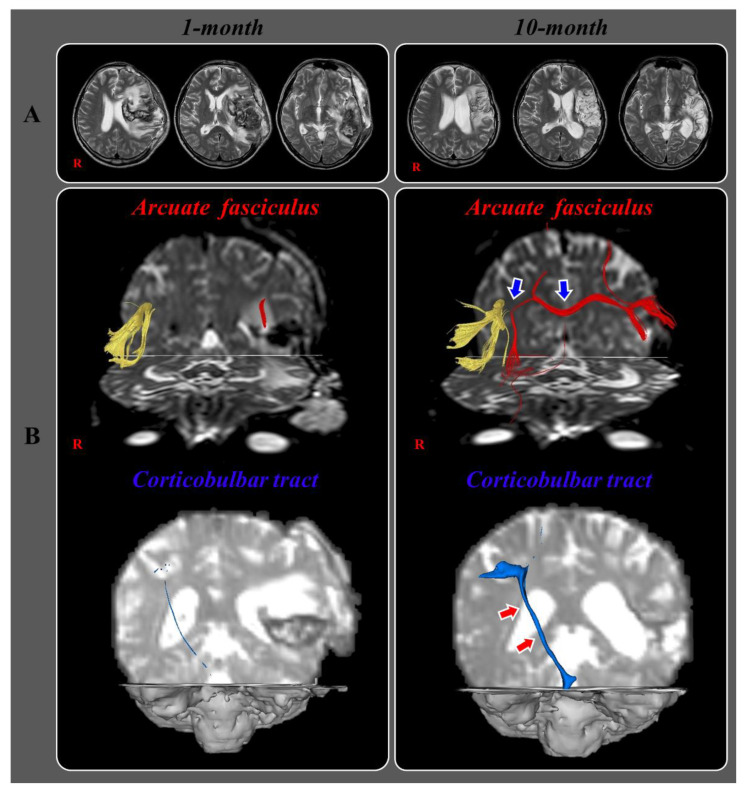
(**A**) Brain MR images at 1 month after onset show an infarct in the left middle cerebral artery territory, hemorrhagic transformation and subfalcine herniation. Brain MR images at 10 months after onset reveal leukomalactic lesions in the left fronto-parieto-temporo-occipital areas (R: right). (**B**) Results of diffusion tensor tractography (DTT) for the arcuate fasciculus (AF) and corticobulbar tract (CBT). On 1-month DTT, the discontinuation of the left AF and severe narrowing of the right CBT are observed. By contrast, on 10-month DTT, the left AF is connected to opposite AF by a new tract that passed through the splenium of corpus callosum (blue arrows) and the right CBT become thicker (red arrows) (reprinted with permission from Ref. [28]).

In 2021, Jang et al. reported a stroke patient who presented with an improvement of aphasia via the transcallosal fibers from the injured left AF to the right corticobulbar tract [28]. The 44-year-old right-handed male patient was diagnosed with an infarction in the left middle cerebral artery territory and received decompressive craniectomy and extra-ventricular drainage for hemorrhagic transformation and brain swelling. Leukomalactic lesions were observed in the left fronto-parieto-temporo-occipital areas on brain magnetic resonance imaging (MRI) at one month after extra-ventricular drainage (Figure 4A). He received rehabilitation including speech therapy at a university hospital and a local rehabilitation hospital for nine months subsequently. The patient showed improvements in moderate conduction aphasia with the AQ of the WAB at ten months after onset compared to one month after onset (total: 46.5 percentile to 49 percentile, spontaneous speech: 35.0 percentile to 71.0 percentile, auditory comprehension: 36.0 percentile to 52.0 percentile, and naming: 53.1 percentile to 59.0 percentile) [58]. On the one-month DTT, the discontinuation of the left AF and severe narrowing of the right corticobulbar tract which appeared to be injured by subfalcine herniation were observed. In contrast, the left corticobulbar tract was not reconstructed. On the ten-month DTT, the left AF was connected to the right AF via transcallosal fibers which passed through the splenium of the corpus callosum, and the right corticobulbar tract had become thicker, while the left corticobulbar tract was not reconstructed (Figure 4B). The authors suggested that the improvement of aphasia in this patient (AQ, one month: 35.0 percentile → ten months: 71.0 percentile) appeared to be attributed to the new language pathway (the left AF → transcallosal fibers → right AF → right corticobulbar tract) following the injuries to the AF and corticobulbar tract in the left hemisphere because the left corticobulbar tract was not reconstructed on the 10-month DTT. As a result, the authors concluded that these results suggest a recovery mechanism of the injured AF and corticobulbar tract in stroke patients. The advantage of this study is that the authors suggested a detailed recovery mechanism following severe injuries to the left AF and corticobulbar tract. The limitation of this study is that it is a case study that reported without the DTT parameter data.

### 4.3. Recovery via the Neural Tracts in the Non-Dominant Hemisphere

In 2009, Schlaug et al. investigated the effect of intonation-based speech therapy on the right AF in chronic stroke patients with aphasia [18]. Six right-handed patients with moderate to severe nonfluent aphasia due to a left hemispheric stroke were recruited at least one year after onset. The number of Correct Information Units (CIU)/min produced during a spontaneous speech, picture descriptions, and descriptions of common procedures was mainly used for the evaluation of aphasia [63]. All the patients underwent Melodic Intonation Therapy which was based on the phenomenon that even patients with severe aphasia can often produce well-articulated, linguistically accurate words while singing, but not during speech (1.5 h/day, five days/week, usually 75–80 or more sessions) [64,65]. The number of CIUs was assessed prior to therapy, after 75 therapy sessions, and again one month later. All patients revealed a significant improvement in CIUs while eliciting spontaneous speech through conversations and descriptions of complex pictures as well as common procedures. DTIs were taken three times for each patient (twice before speech therapy, and once after speech therapy). Only the right AF was reconstructed because the left AFs were not fully identified in each of the six patients. The fiber number of the right AF was significantly increased in the post-therapy DTT compared to the pre-therapy DTT in all the patients. However, the two pre-therapy DTTs did not show any significant difference. The change in the fiber number of the right AF revealed a strong trend with a change in CIU/min without significant difference (*r* = 0.7; *p* = 0.1). The authors determined a ratio between the fiber numbers of the AF and the corticospinal tract (CST) to normalize the pre-and the post-therapy DTT results and observed coinciding results. As a result, the authors suggested that Melodic Intonation Therapy could lead to a change in the right AF. The advantage of this study was that the authors clearly demonstrated the effect of the speech therapy program by showing no interval change between the two pre-therapy DTTs and normalization compared with the changes in the CST. However, the authors analyzed only the right AF and did not provide other DTT parameters such as the FA and mean diffusivity.

In 2020, Blom-Smink et al. investigated the changes of the language-related neural tracts in the dorsal and ventral language white matter tract in relation to naming recovery in subacute stroke patients with aphasia [26]. Ten right-handed patients with moderate aphasia due to a left-hemisphere stroke (infarction: eight patients, and hemorrhage: two patients) were recruited. The BNT and DTI were performed twice within three months after onset with a one-month interval (the first DTI: mean 39.6 days after onset; and the second DTI: mean 79.1 days after onset) [57]. All patients underwent a stroke rehabilitation program, including speech therapy which comprised an intensive naming therapy based on the Cueing Hierarchy Therapy (CHT) [66], cognitive-linguistic therapy, communicative treatment, and/or counseling and coaching of patients and proxies, that was performed for two weeks (45 min/day). Six patients presented improvement on the BNT (5~15 points), two patients revealed limited improvement (1 and 2 points, respectively), and two participants showed a slight decline (−1 and −3 points, respectively). The superior longitudinal fasciculus (SLF), inferior fronto-occipital fasciculus (IFOF), inferior longitudinal fasciculus (ILF), middle longitudinal fasciculus (MLF), and uncinate fasciculus (UF) were reconstructed, and the FA value was determined for each tract. A strong positive correlation was found only between the change of the FA value of the right ILF and the change in the BNT (*r* = 0.91, *p* < 0.001). Consequently, they concluded that naming recovery in subacute aphasia of stroke patients is associated with a change in the integrity of the right ILF. The advantage of this study is that most of the language-related tracts were analyzed except for the AF. However, the other DTT parameters such as the mean diffusivity value and fiber number were not determined. The relatively small number of subjects was a limitation of this study. In addition, the relationship of the stroke lesions with the left language-related neural tracts was not provided and the lesion volume was quite variable (5.57~154.57 mL). Furthermore, the variable evaluation intervals between patients and the fact that some of the patients underwent tDCS were the other limitations of this study.

## 5. Conclusions

In this mini-review, twelve previous DTT-based studies on the recovery mechanisms of aphasia in stroke were reviewed. We classified the twelve studies into the following three categories according to the recovery mechanisms: recovery via the neural tracts in the dominant hemisphere (eight studies), via transcallosal fibers (two studies), and via the neural tracts in the non-dominant hemisphere (two studies) [18,19,21,22,23,24,25,26,27,28,29,30]. Ten of the twelve studies were conducted for the AF although many other neural tracts are known to be involved in language processing. Also, eight of the above ten studies were focused on the AF in the dominant hemisphere [18,19,21,22,23,24,27,28,29,30]. An analysis of these studies reveals that only one mechanism of aphasia recovery in stroke (restoration of the integrity of the injured AF in the dominant hemisphere) was clearly demonstrated by previous studies. As with various motor recovery mechanisms which have been most actively researched in stroke—for example, recovery of the injured corticospinal tract (recovery by restoration of normal integrity, transcallosal fibers, aberrant pyramidal pathway, peri-lesional reorganization, and compensation by the corticoreticulospinal tract)—there could also be other recovery mechanisms of aphasia in stroke patients because more neural tracts are known to be involved in language processing than in motor execution [37,38,39,40,41,42,44,45,67,68,69,70]. However, based on our review of available data, very little is known about the recovery mechanisms of aphasia in stroke because of the following reasons: first, a paucity of studies on the subject— only twelve studies, and seven of the twelve studies were case reports [18,19,21,22,23,24,25,26,27,28,29,30]; second, the majority of the twelve studies analyzed only some of the language-related neural tracts or the AF in the dominant hemisphere [18,19,21,22,23,24,25,27,28,29,30]; third, there were only two studies on the contribution of the language-related neural tracts in the non-dominant hemisphere [18,26]. Therefore, further original studies involving a larger number of patients with aphasia in stroke should be encouraged forthwith. Further studies involving various lesion locations and severity levels of injuries of the language-related neural tracts should also be conducted because the recovery mechanisms of aphasia in stroke could be dependent on these factors. In addition, combined studies with functional neuroimaging studies could be helpful in elucidating the recovery mechanisms which are related to the activations of the peri-stroke area and the homologous language areas in the nondominant hemisphere [8,11,12,13,14,15,16,17].

## Figures and Tables

**Table 1:** Correlation between the language subdomains and the fractional anisotropy of the language-related neural tracts reported by Zhang et al. [45].

		Language Subdomains				
		Naming	Repetition	Syntactic Processing	Reading	Comprehension
Left dorsal stream	AF	-	0.48 *	0.61 *	-	-
	SLF	-	-	0.55 *	-	-
Left ventral stream	IFOF	0.56 *	-	-	0.34 *	0.53 *
	UF	0.49 *	-	-	0.28 *	0.38 *
	ILF	0.35 *	-	-	-	0.48 *

Values indicate correlation coefficient. AF: arcuate fasciculus, SLF: superior longitudinal fasciculus, IFOF: inferior fronto-occipital fasciculus, UF: uncinate fasciculus, ILF: inferior longitudinal fasciculus. * Significant correlation (*p* < 0.05).

**Table 2:** Diffusion tensor tractography studies on the mechanisms of aphasia recovery in stroke.

Authors	Publication Year	Patient No.	Stroke Type	Duration to 1st DTI	Aphasia Evaluation Method	Analyzed Neural Tracts	Results
Recovery via the neural tracts in the dominant hemisphere							
Breier et al. [19]	2011	1	Infarction	5 years	AQ of WAB	AF	FA ↑ (Lt. AF)
van Hees et al. [22]	2014	8	Not described	17~170 months	BNT	AF UF	FA ↑
Jang and Lee [21]	2014	1	Hemorrhage	1 month	AQ of WAB	AF	Elongation (Lt. AF)
Nunnari et al. [23]	2014	1	Infarction	Not described	Neuropsychological Examination of Aphasia Battery	AF	FA ↑ Fiber number ↑ (Lt. AF)
Jang et al. [24]	2017	16	Infarction: 11 Hemorrhage: 5	Within 30 days	AQ of WAB	AF	Voxel number (Lt. AF): positive correlation with AQ
Kieronsk et al. [29]	2021	1	Infarction	1 day	FAST BNT	AF UF	Fiber number ↑ Tract volume ↑ (Both AFs and Lt. UF)
Choi et al. [27]	2021	1	Hemorrhage	8 weeks	AQ of WAB	AF	Elongation FA ↓ Fiber number ↑ (Lt. AF)
Bae et al. [30]	2022	35	Stroke	Average 36.06 days	WAB	AF	FA (Lt. AF): positive correlation with AQ
Recovery via the transcallosal fibers							
Yu et al. [25]	2018	1	Infarction	14 months	Modified WAB	Corpus callosum	Transcallosal fibers ↑ (to Lt. Werniche’s and Rt. Broca’s areas)
Jang et al. [28]	2021	1	Infarction	1 month	AQ of WAB	AF	Transcallosal fiber ↑ (to Rt. AF)
Recovery via the neural tracts in the non-dominant hemisphere							
Schlaug et al. [18]	2009	6	Not described	1 year	Correct information units	Rt. AF	Fiber number ↑ (Rt. AF)
Blom-Smink et al. [26]	2020	10	Infarction: 8 Hemorrhage: 2	Average 39.6 days	BNT	SLF IFOF ILF MLF UF	Change of FA of Rt. ILF: positive correlation with change of BNT

DTI: diffusion tensor imaging, AQ: Aphasia Quotient, WAB: Western Aphasia Battery, AF: arcuate fasciculus, FA: fractional anisotropy, BNT: Boston Naming Test, UF: uncinate fasciculus, FAST: Frenchay Aphasia Screening Test, SLF: superior longitudinal fasciculus, IFOF: inferior fronto-occipital fasciculus, ILF: inferior longitudinal fasciculus, MLF: middle longitudinal fasciculus.

## Data Availability

Not applicable.
